# The Bahraini food based dietary guidelines: a holistic perspective to health and wellbeing

**DOI:** 10.3389/fpubh.2023.1182075

**Published:** 2023-06-12

**Authors:** Farah Naja, Sharfa Khaleel, Maryam Ebrahim Alhajeri, Buthaina Yusuf Ajlan, Najat Mohamed Abulfateh, Amna Ghassan Alawadhi, Marwa Husain Jan Bowah, Ayoub Al-Jawaldeh

**Affiliations:** ^1^Department of Clinical Nutrition and Dietetics, College of Health Sciences, Research Institute of Medical and Health Sciences (RIMHS), University of Sharjah, Sharjah, United Arab Emirates; ^2^Department of Nutrition and Food Sciences, Faculty of Agricultural and Food Sciences, American University of Beirut, Beirut, Lebanon; ^3^Ministry of Health, Sanabis, Bahrain University of Beirut, Beirut, Lebanon; ^4^Regional Office for the Eastern Mediterranean (EMRO), World Health Organization (WHO), Cairo, Egypt

**Keywords:** food-based dietary guidelines, biopsycho-ecological theory, sustainability, food waste, Bahrain

## Abstract

The impact of food consumption extends well beyond the physical aspect of health to affect the mind, the society, and the environment. The biopsycho-ecological (BSE) theory recognizes the interplay between these factors and emphasizes the need for a holistic perspective to dietary recommendations. This manuscript presents a situation analysis of food consumption and diet-related diseases in Bahrain and describes the themes of the Bahraini Food based dietary guidelines (FBDG) and their alignment with the BSE constructs. Available data revealed low fruit and vegetable intake and excessive consumption of processed meat and sugary drinks in the country. These dietary habits are accompanied by a high burden of non-communicable diseases and their risk factors, anemia, and vitamin D deficiency. The Bahraini FBDG consisted of 11 context-specific themes and key messages that addressed the four dimensions of health depicted by the BSE theory, as follows: diet, physical activity, and food safety (body), physical activity, mindful eating and mental health (mind); family relations and cultural heritage (society), and food waste and environmental footprints of dietary intake (environment). The Bahraini FBDG present a model of dietary guidelines that adopted a holistic perspective to address health as they promote the role of food and dietary habits in maintaining the health of the body and that of the mind, the society, and the environment.

## 1. Introduction

In the 20th century, health and wellbeing were primarily understood through the scientific discipline of pathophysiology, which focused on the biochemical and physiological mechanisms that informed disease prevention, diagnosis, and treatment (1). However, this reductionist approach failed to explain many diseases, leading to the development of the biopsychosocial model by Engel in 1977. This model attributed health and disease to the complex interactions between biological, psychological, and social factors (2). More recently, Stineman et al. proposed the biopsycho-ecological (BSE) model, adding the environment as a fourth interacting dimension to the biopsychosocial model. The BSE model recognizes that human health is an integral part of a larger ecosystem and that the environment interacts closely with the body, mind, and society (3). The BSE model of health was reinforced following the release of the Sustainable Development Goals (SDGs), in 2015, which coined the health of the environment as an integral component to human health (4).

Dietary intake has long been recognized as an important factor that impacts health and wellbeing. Initially, the focus was on disease prevention and management, but scientific evidence now shows that food intake is related to all aspects of health, including mental and social health. Recently, the SDGs presented a framework for the effect of dietary intake on the health of the environment. Within this framework, dietary intake is directly implicated in SDG 6 (Clean Water and Environment), SDG 13 (Climate Action), SDG 14 (Life Below Water), and SDG 15 (Life on Land). Food production and consumption accounts for up to 30% of (GHG) emissions, uses 70% of freshwater use, and occupies more than a third of all potentially cultivatable land. In fact, emerging evidence indicates that the adoption of sustainable dietary habits can enhance environmental health by decreasing the strains on the use of natural resources, including the water, energy and land needed for food production. In addition, sustainable consumption patterns can reduce the amount of food loss and food waste, both of which are main pillars of the SDGs (5, 6). Given this multi-dimensional impact, dietary recommendations are expected to reach beyond physical health alone and address mental, social, and environmental health as well. In this context, the BSE model, which consists of body, mind, society, and environment could constitute a suitable framework to formulate food and diet recommendations that address health using a holistic approach.

The aim of this manuscript is to introduce the Bahraini Food Based Dietary Guidelines (FBDG) as a model of dietary and food recommendations that addresses health using a holistic perspective, following the BSE theory, hence accounting for the body, mind, society as well as the environmental aspects of health. Specific objectives include (1) presenting a situation analysis of food consumption and diet related diseases in Bahrain; (2) describing the 11 key messages of the Bahraini FBDG, and (3) highlighting the constructs of the BSE within the guidelines.

## 2. Situation analysis: food consumption, dietary habits, and diet-related diseases in Bahrain

Bahrain is one of the six countries of the Gulf Cooperation Council (GCC). Following the discovery of oil in the 1970’s, Bahrain has witnessed a marked economic and financial development that has resulted in major changes in the diet and lifestyle of its population.

### 2.1. Food consumption and dietary habits

Similar to many countries of the GCC, the food consumption in Bahrain is marked by an overconsumption of harmful foods coupled with low intakes of protective foods. For instance, 85% of Bahraini adults consumed less than the recommended amounts of fruits and vegetables (<400 g/d) (7, 8). The consumption of milk, nuts and seeds, whole grains, and omega-3 (seafood) were also insufficient (76.18 g/d, 8.64 g/d, 7.18 g/d, and 62.74 mg/d, respectively) (9), falling below their cut-offs of 240 g/d, 16 g/d, 125 g/d, and 250 mg/d, respectively (10). Three out of four adults in Bahrain (76%) did not consume enough olive oil, and 32% of adults did not use it entirely (11). On the other hand, red meat and processed meat intakes were relatively high (160 g/week and 4.38 g/d, respectively) (9), with values greater than the recommended (100 g/week and 0 g/day, respectively) (10). Soft drinks were consumed by 67% of adults, of who 54% used sugar sweetened soft drinks (11).

Available data showed sub-optimal physical activity levels among adults including that practiced at home, at work, and of various intensities. The high prevalence of insufficient physical activity (< 150 min per week) was higher among females as compared to males (7). While no data existed on breakfast skipping and fast-food consumption among adults in Bahrain, available data showed that these lifestyle habits were quite prevalent among adolescents in the country. An alarming proportion of 56% of adolescents did not consume breakfast regularly and 14.4% of adolescents reported eating fast foods on a daily basis (12). These habits are of major concern especially in this age group, where dietary habits tend to be formed and persist until adulthood (13).

Related to the sustainability aspect of food consumption are food waste and environmental footprints (EFPs) associated with dietary intake. In terms of food waste, Bahrain ranked first among Arab countries with around 250,000 tons of leftovers thrown out annually (132 Kg/capita/year) (14). Though no data exist to characterize the EFPs associated with food intake in Bahrain, available estimates showed that, in spite of its relatively small population, Bahrain has one of the world’s largest carbon footprints *per capita*, ranking second after Qatar in terms CO2 production (22.3 tons *per capita* compared to the global average of 4.4) (15, 16). Such high EFPs could be in part due to the high consumption of animal products (namely red and processed meat) by adults living in Bahrain. Meat production and consumption exert enormous strains on natural resources while generating different typologies of waste, from food waste to packaging (17). For instance, while food production accounts for 30% of the global CO2 emissions, 60% of it is due to meat production (18). Furthermore, the consumption of meat and animal products makes up 27% of the global total water footprint (19).

### 2.2. Diet-related diseases in Bahrain

Despite major strides in the advancement of the health care system in Bahrain, the country is currently bearing a heavy burden of non-communicable diseases (NCDs), including cardiovascular diseases (CVD) and diabetes and their risk factors (20). In addition to the NCDs, significant prevalence rates of micronutrients deficiencies are also present in the country.

In Bahrain, CVD are one of the primary causes of death, with a proportional mortality of 28% (21). In addition to CVD, the prevalence rate of diabetes in Bahrain was among the highest in the region (15% among adults in year 2018) (7, 22). Of concern is the increasing trend of the prevalence of diabetes in the country (14.3% in 2007 to 15% in 2018) (22). In line with the burden of NCDs was that of their major risk factors, including hypertension, obesity, and hyperlipidemia. Existing data showed that 33.6% of adults in Bahrain were diagnosed with hypertension in 2018, with higher proportions observed among males compared to females (38.7 and 26%, respectively) (7). As for overweight and obesity (BMI >25 kg/m2), their prevalence rates have reached alarming levels (72.4% of adults), with comparable data among males and females (71.7 and 73.4%, respectively) (7). Hyperlipidemia is also among the NCD risk factors that is prevalent in Bahrain, with 22.2% of adults having a high LDL-C (≥ 3.4 mg/dL), 42.2% have high triglycerides (≥ 1.7 mg/dL) and alarming percentage (64.5%) have low HDL-C (≤ 1.3 mg/dL) (7, 23).

Among the micronutrient deficiencies that are of concern in Bahrain are that of iron and vitamin D. In 2003, 51% of women aged 14–49.9 years had hemoglobin (Hb) <12 g/dL. Similar observations were recorded among men, where 21% of those above the age of 19 years had Hb <13 g/dL (24–26). Regarding vitamin D deficiency, though its prevalence in Bahrain is lower than that in other countries of the region, it remains a common health concern, whereby 41.4% of adults in 2018 were considered vitamin D deficient (27–30).

## 3. Description of the Bahraini FBDG

The current food consumption and burden of diet-related health issues in Bahrain triggered national efforts to develop programs and interventions to promote healthy eating and lifestyles, at the core of which are country-specific FBDG. The development of the Bahraini FBDG followed a participatory and evidence-based methodology involving national, international, and technical experts. A triangulated approach was used, consisting of:

An examination of the current situation of the diet- related diseases and food consumption in Bahrain, using published as well as grey literature.

Extensive review and synthesis of cutting-edge and evidence-based research addressing the association of diet with health and diseases, with a focus on the diseases prevalent in Bahrain.

International and local experts’ vetting of the science using a culture and context specific lens.

A mixed-method approach combining content and thematic analyses was used to synthesize the collected information and to derive the guidelines and recommendations. More specifically, a coding scheme referring to the various themes of dietary recommendations, identified by content analysis, was developed. The thematic analysis was then implemented using this coding scheme to systematically review the various documents, assigning similar pieces of information the same code. The panel of experts examined the results of the coding for synthesis and interpretation, identifying, ranking, and prioritizing the themes that are most relevant within the context of Bahrain. As such, the Bahraini FBDG consisted of 11 themes and corresponding key messages (Table 1). The guidelines encouraged maintaining a healthy body weight; enjoying a diverse diet rich in fruits, vegetables, and whole grains; minimizing red meat, processed food, sugar, and salt; and increasing the consumption of plant proteins such as legumes and nuts. In addition, the guidelines promoted specific behaviors such as exercise, reducing food waster, participating in family meals, mindful eating and connecting with one’s culture and heritage. Given the public health orientation of the guidelines, they were not intended to replace any clinical guidelines for treating chronic diseases. As such, the Bahraini FBDG targeted mainly healthy adult population. People requiring special diets were advised to consult with a dietitian or other health professional for more guidance.

**Table 1:** Themes and corresponding key messages of the Bahraini FBDG.

	Themes	Key messages
1	Healthy body weight	Look after your body weight today to enhance your health tomorrow
2	Physical activity	Move more. Exercise not only optimizes your body, it enhances your mind and mood
3	Varied/balanced diet	Try varying and balancing your diet. This a lifestyle, not “all or nothing”
4	Fruits and vegetables	Keep it simple by sticking to more fruits and vegetables
5	Healthy animal proteins	Switch to healthier animal proteins, while emphasizing low fat milk and dairy and fish/sea food
6	Vegetarian alternatives	Choose vegetarian alternatives to red and processed meat such as legumes and unsalted nuts
7	Sugar and salt	Pay attention to your intake of salt and sugar, especially the hidden ones
8	Water and fluids	Stay hydrated with water and healthy fluids
9	Food safety	Observe the recommendations for safe food production and consumption
10	Food sustainability	Contribute to protecting the environment, feeding the hungry and saving money by decreasing food waste
11	Homemade meals and mindful eating	Eat homemade foods with others to promote enjoyment and connect with cultural heritage

## 4. Alignment of the Bahraini FBDG with the constructs of the BSE

By design, the Bahraini FBDG aimed to address health holistically. As such, together the 11 guidelines addressed the four dimensions of the BSE health model: the body, the mind, the society as well as the environment.

### 4.1. Physical health (body)

Throughout most of Bahraini guidelines, physical health has been addressed. The first guideline encourages maintaining a healthy body weight as a determinant not only of current health status but also in the future. A review of 239 prospective studies in four continents showed that the associations of both overweight and obesity with higher all-cause mortality were broadly consistent (31). On the other hand, being underweight increased the risk of nutrient deficiencies, anemias, bone loss, and early mortality (32). Two of the Bahraini FBDG underscored the importance of choosing plant-based foods, specifically encouraging the consumption of fruits, vegetables, legumes and nuts. Plant-based diets have been consistently linked to health benefits, such as lower risk of cancer, type 2 diabetes, and CVD (33). Guideline 6 (vegetarian alternatives) implied replacing the animal sources of proteins with those of plant origin. In fact, a meta-analysis of RCTs suggested that substituting red meat with high-quality plant protein sources was shown to affect favorably blood lipids and the risk for metabolic abnormalities (34). Limiting sugar and salt was the only guideline that had a negative wording, given the importance of the reduction of salt and sugar on one’s health and the overwhelming evidence for the risks associated with their higher consumption. A recent review found that a 100 mmol/d increment in sodium intake was associated with a significantly higher risk of both CVD incidence and mortality (35). Similarly, a higher sugar intake is strongly implicated in the etiology of many diseases including diabetes, CVD as well as cancer (36).

In addition, the Bahraini FBDG addressed lifestyle exposures such as physical activity, hydration and food safety, as integral elements of physical health. The evidence for the positive effects of physical activity on disease prevention is overwhelming (37). For instance, a meta-analysis of 1,616 studies showed that aerobic physical activity could be recommended as a form of treatment for cancer, CVD as well as diabetes (38). The guideline targeting hydration within the Bahraini FBDG had two main messages: underscoring the importance of drinking water and encouraging the consumption of healthy fluids. The latter inferred limiting drinking ‘less healthy’ fluids mainly the sugar sweetened beverages (SSBs). Drinking enough water is essential for physical health, as it is involved in most body functions (39–41). This is particularly relevant for people living in countries with hot weather such as Bahrain. Lastly, observing recommendations for safe food consumption and production is an essential element of how diet could influence physical health. According to the WHO, each year worldwide, consumption of unsafe food leads to 600 million cases of foodborne diseases and 420,000 deaths, most of which are preventable (42).

As such, building on the latest scientific evidence and guided by available data on food consumption and diet related diseases in the country, 9 out of the 11 Bahraini FBDG directly targeted the health of the body (Table 2).

**Table 2:** Alignment of the Bahraini FBDG with the domains of the BSE model of health.

	Body	Mind	Society	Environment
Look after your body weight today to enhance your health tomorrow	✓			
Move more. Exercise not only optimizes your body, it enhances your mind and mood	✓	✓		
Try varying and balancing your diet. This a lifestyle, not “all or nothing”	✓			
Keep it simple by sticking to more fruits and vegetables	✓	✓		✓
Switch to healthier animal proteins, while emphasizing low fat milk and dairy and fish/sea food	✓			
Choose vegetarian alternatives to red and processed meat such as legumes and unsalted nuts	✓	✓		✓
Pay attention to your intake of salt and sugar, especially the hidden ones	✓			
Stay hydrated with water and healthy fluids	✓			
Observe the recommendations for safe food production and consumption	✓			
Contribute to protecting the environment, feeding the hungry and saving money by decreasing food waste			✓	✓
Eat homemade foods with others to promote enjoyment and connect with cultural heritage		✓	✓	

### 4.2. Mental health (mind)

In addition to physical health, many of the Bahraini FBDG included messages targeting mental health. First the Bahraini FBDG explicitly addressed the health of the mind ‘Exercise not only optimizes your body, it enhances your mind and mood’. The positive effects of exercise on mental health has been often neglected, despite the accumulating evidence for it being an effective intervention to reduce depression/anxiety and improve self-perception (43). Second, by referring to enjoyment of food in the last guideline, the Bahraini FBGD supported mindful eating. The latter has been shown to correlate positively with mental well-being (44). In addition to these two guidelines that directly targeted mental health, certain dietary recommendations within the Bahraini FBDG may also positively influence mental health. In fact, according to a meta-analysis of randomized controlled trials addressing the effects of dietary modifications on mental health, dietary interventions were found to be promising novel approach to alleviate the symptoms of many mental disorders including depression (45). More specifically, the literature suggests that a diet rich in fruits, vegetables, fish and whole grains is associated with reduced risk of anxiety and depression (46). For instance, fruits and vegetables are considered rich sources of antioxidants (e.g., vitamin C, E, beta-carotene) and polyphenols (e.g., flavonoids, phenolic acids). These antioxidants lower oxidative stress and ultimately reduce chronic inflammation in the body. The latter has been implicated in the etiology of anxiety and depression. In addition, these foods are high in fiber, which promotes the growth of beneficial gut bacteria, also known to reduce the risk of anxiety and depression. Furthermore, they are rich in various vitamins, minerals, and complex carbohydrates needed to support brain function and regulate mood (47).

### 4.3. Social health (society)

Acknowledging the fact that eating is largely considered a social act, the Bahraini FBDG specifically underscored the importance of eating meals with family and friends. The social gatherings around meals reinforces social ties and networks. Family meals provide opportunities for communication, positive engagement, sharing of values and family bonding (48). A central feature of common values shared in family meals, in a country where most inhabitants are of Muslim faith, is the concept of ‘Halal’ foods (Halal which means lawful or permitted according to Islamic principles). It has been argued that Halal food production and consumption, based primarily on biologic agriculture, fair trade, and green animal breeding, may have positive effects not only at the individual but also at the societal levels (49).Within the same guideline that addressed eating with family and friends, recommendation to eat home cooked meals was also included. Consuming homemade food play an important role in connecting individuals among each other and to their cultural heritage hence further contributing to social health (50). Furthermore, by encouraging sharing food with the less fortunate, the Bahraini FBDG aimed to improve social cohesion, a main determinant of social health. Social cohesion has been increasingly recognized as a vehicle for sustainable poverty reduction as a mean to decrease the sentiments that the fruits of growth are not equally shared (51). In addition to the aforementioned direct links between two of the Bahraini FBDG and social health, the rest of guidelines may also be indirectly implicated in this regard. By improving physical and mental health, these guidelines contribute to a better self-esteem, enabling the individual’s social confidence and strengthening the desire to forge new relationships and social connections (52).

### 4.4. Environmental health (environment)

Following the release of the SDGs in 2015, most of the dietary guidelines were revised to address not only physical health but also the various sustainability dimensions, including the economic, social as well as environmental (53). The quality of the food and the dietary choices as well as food waste are tightly linked to environmental footprints (17). The current dietary habits in Bahrain are marked by high consumption of red and processed meat and low intake of fruits and vegetables. Both of these traits of food consumption lead to high CO2 emissions, water and energy use. A systematic review showed that shifting from a typical western diet, based on meat and meat products, to a more plant-based diet, rich in fruits and vegetables may reduce 70% of GHG emissions and land use, and 50% of water use associated with food consumption (54). As such, by recommending plant-based alternative to animal proteins, the Bahraini FBDG not only promote foods that are healthy for humans but also that reduce the impact of food consumption on the environment. Furthermore, in the context of the environmental sustainability, food waste accounts for one-third of all human-caused GHG emissions and generates 8% of GHG gases annually (55). Therefore, the Bahraini FBDG called for a decrease in food waste, especially with the significant food waste reported in Bahrain which ranks first among Arab countries in terms of food waste (14).

## 5. Conclusion

In a country with surging rates of most diet-related diseases, a considerable prevalence of suboptimal dietary habits, high food waste and large EFP, it was warranted for the FBDG of the Bahrain to reach beyond the physical health and address the mental, social as well as the environmental health aspects of the population. Using the BSE as a framework for a holistic approach to health and wellbeing, the 11 messages of the Bahraini FBDG aimed to optimize the role of diet in fostering a better health of the body, mind, society as well as the environment. Though the Bahraini FBDG are designed to address Bahraini adults and have limited generalizability to other countries, the holistic approach for health used in these guidelines may serve as a model for other nutrition as well as lifestyle recommendations.

## Data availability statement

The original contributions presented in the study are included in the article/supplementary material, further inquiries can be directed to the corresponding author.

## Author contributions

FN and AA-J: conceptualization. FN: methodology and writing—original draft preparation. SK: formal analysis. MA and BA: investigation and supervision. NA and AA: resources. AA-J, MB, and SK: writing—review and editing. All authors have read and agreed to the published version of the manuscript.

## Conflict of interest

The authors declare that the research was conducted in the absence of any commercial or financial relationships that could be construed as a potential conflict of interest.

## Publisher’s note

All claims expressed in this article are solely those of the authors and do not necessarily represent those of their affiliated organizations, or those of the publisher, the editors and the reviewers. Any product that may be evaluated in this article, or claim that may be made by its manufacturer, is not guaranteed or endorsed by the publisher.
